# Household inhalants exposure and nasopharyngeal carcinoma risk: a large-scale case-control study in Guangdong, China

**DOI:** 10.1186/s12885-015-2035-x

**Published:** 2015-12-29

**Authors:** Yong-Qiao He, Wen-Qiong Xue, Guo-Ping Shen, Ling-Ling Tang, Yi-Xin Zeng, Wei-Hua Jia

**Affiliations:** State Key Laboratory of Oncology in South China, Collaborative Innovation Center for Cancer Medicine, Sun Yat-Sen University Cancer Center, 651 Dongfengdong Road, Guangzhou, Guangdong 510060 China; Department of Radiation Oncology, The First Affiliated Hospital, Sun Yat-sen University, Guangzhou, 510060 China

**Keywords:** Incense burning, Mosquito coil, Cooking fumes, Wood fuel using, NPC risk

## Abstract

**Background:**

Epidemiological studies show that cigarette smoking increase the risk of nasopharyngeal carcinoma (NPC), however, whether other common, potentially adverse household inhalants increase NPC risk remains uncertain.

**Methods:**

We conducted a large case-control study to explore the effects of household inhalants, such as incense, mosquito coil, cooking fumes, and wood combustion, on NPC risk. We recruited 1,845 cases and 2,275 controls from Guangdong province, a high-risk area for NPC in China, to obtain the demographic data and relevant exposure information through face-to-face interviews.

**Results:**

We found that incense burning was associated with NPC risk by comparing frequent incense use with never using incense [OR and 95 % confidence interval (CI) = 1.73, (1.43, 2.09)]. Wood fuel use was also associated with NPC risk compared with non-wood fire use [OR and 95 % CI = 1.95, (1.65, 2.31)]. More intriguingly, we observed a significant addictive interaction between frequent incense burning and heavy cigarette smoking on NPC risk [synergistic index (SI) = 1.67; 95 % CI: 1.01, 2.76]. We also found a significant joint effect between wood fuel use and NPC family history for NPC risk (SI = 1.77; 95 % CI: 1.06, 2.96). However, neither mosquito oil nor cooking fumes were associated with NPC risk.

**Conclusions:**

Our study shows that incense smoke is not only the potential independent risk factor but also co-contributes with cigarette smoking to NPC risk. Moreover, wood combustion is another potential environmental risk factor and exerts a joint effect with NPC family history on NPC.

**Electronic supplementary material:**

The online version of this article (doi:10.1186/s12885-015-2035-x) contains supplementary material, which is available to authorized users.

## Background

According to the latest WHO report, there were 4.3 million deaths in 2012 due to household air pollution globally, which reflects a large increase over the estimated 2 million deaths in 2004 (http://www.who.int/phe/health_topics/outdoorair/databases/FINAL_HAP_AAP_BoD_24March2014.pdf?ua=1). This is especially an issue for Southeast Asia, where environmental issues have become increasingly prominent and which bears the greatest share of the burden worldwide at 1.69 million deaths. Short-term effects of indoor air pollution can cause acute mucosal irritation of the eyes, nose and throat. For long-term exposure, indoor air pollution can lead to pneumonia, stroke, ischemic heart disease, chronic obstructive pulmonary disease (COPD), lung cancer and other issues. Most people spend more than half of their lives in the house, so it is extremely urgent to pay ample attention to the health effects of household air pollution.

Nasopharyngeal carcinoma (NPC) shows a distinctive geographic distribution, with an incidence of 20–50 per 100,000 in southern China and southeast Asia compared to most of the world, where it is a rare occurrence [1–3]. Its apparent racial clustering and regional differences indicate that genetic traits play a large role in the pathogenesis of NPC. Furthermore, we have seen a decreasing incidence of NPC in some high risk areas in recent decades, likely due to changes in traditional lifestyles and enhanced health consciousness, such as lower consumption of preserved food and salted fish, a decline in cigarette smoking, the increasing westernization of dietary habits, and early screening for EBV antibodies, which suggest that external environmental factors are significant as well in the occurrence of NPC and are increasingly capturing people’s attention [4–7].

For centuries, Buddhism and Taoism have been the principal religions in Southeast Asia, with approximately half of the populations paying homage to deities for good fortune through the traditional practice of incense burning. Burning incense releases enormous quantities of fine particulate matters and high concentrations of harmful gases and volatile organic compounds. Previous studies have indicated that incense compounds include several mutagenic and genotoxic materials, such as formaldehyde and carbonyls, which greatly influence the environment and would be inhaled by those in the vicinity [8, 9]. Researchers have conducted studies to assess the association between incense burning and NPC risk in Hong Kong and Singapore with inconsistent results. Several case-referent studies performed in Hong Kong found a positive effect of incense burning on NPC risk [10–12], while the only population-based cohort study to date, conducted by Friborg et al. found a null association between incense burning and NPC risk among Singapore Chinese [13].

It is estimated that nearly 40–50 billion mosquito coils are consumed worldwide each year by almost 2 billion people to repel mosquitos, which are a nuisance and carry diseases [14]. According to a large-scale survey conducted in Shanghai, China, more than half of individuals use mosquito-repellent at home [15]. Mosquito coils, which mainly consist of the active ingredient pyrethrum combined with biomass base materials, emit insecticides fumes to prevent mosquitos from biting through slow and steady combustion. Reports have indicated that burning mosquito coils can release large amounts of fine particles, polycyclic aromatic hydrocarbons (PAHs), volatile organic compounds (VOCs), and carbonyl compounds and can have immediate and long-term health effects [16, 17]. Burning mosquito coils has been demonstrated to have a strong positive association with respiratory diseases, such as chronic obstructive pulmonary disease and lung cancer [18–20].

Cooking oil fumes are another common, everyday household inhalant that includes more than two types of carcinogens [21]. Furthermore, studies have demonstrated that cooking fumes can induce a type of apoptosis of protein inhibitors that participate in lung cancer cell survival and proliferate to increase the risk of lung cancer [22–26]. Additionally, cooking at high temperatures, poor ventilation and using certain fuel types will increase lung cancer risk [22, 27, 28]. In addition, the use of wood fuel for cooking and heating, which may release quantities of fine particulates and harmful fumes, is considered to be a potential adverse inhalant. It is estimated that almost 2 million people die globally every year from using solid fuel, and several studies have been conducted to assess the association between wood fuel use and NPC risk among Guangxi Chinese [29] and Singapore Chinese [30]. However, these studies have been limited by small sample size and inconsistent results.

Hazardous indoor inhalants, such as incense burning, mosquito coils, cooking fumes and wood combustion, which contain numerous potentially harmful substances, are inhaled into the body and can lead to acute or chronic health issues. As the nasopharynx is the first place where these unhealthy inhalants enter the body, it is important to determine whether these indoor inhalants will result in long-term harm to the nasopharynx, including causing tumors.

Given the inadequate epidemiological evidence for the association between potentially harmful household inhalants such as incense burning, mosquito coils, cooking fume and wood fuel exposures and NPC in high-risk areas of southern China, we performed a case-control study to investigate the association between these household air pollutants and NPC risk.

## Methods

The case-control study described in this paper has been previously reported in detail [31–34]. In summary, pathologically diagnosed NPC cases were recruited from the Sun Yat-Sen University Cancer Center, the largest cancer prevention and treatment center in southern China, between October 1, 2005 and October 1, 2007. Meanwhile, healthy controls from all 21 municipalities in the Guangdong province were enrolled from the general hospital’s physical examination center and frequency-matched by sex and age (±5 years). All of those recruited were local residents who had lived there for at least 5 years and were able to complete the interview. An informed consent was obtained from every subject before the interview, and our study was approved by the human ethics committee of the Sun Yat-Sen University Cancer Center. In total, 1948 electable NPC cases were identified and 1845 (94.7 %) completed the interview. Of the 2381 healthy candidates, 2275 (95.5 %) eligible controls finished the questionnaire and were enrolled in our study as well. The main reason for the drop-out of 103 cases and 106 controls was refusal to complete the questionnaire. Well-trained investigators administered to every subject through face-to-face interviews by well-designed, structured questionnaires which have been previously reported. The collected information for social demographic characteristics includes items such as age, sex, education level and so on.

We defined those who smoked at least one cigarette every 3 days for at least 6 months as smokers, including current smokers and ex-smokers; those who smoked more than 20 pack-years were defined as heavy smokers. For incense burning, participants were asked to choose from four categories of burning incense frequency: never burn incense, burn incense during festivals, burn incense on the first and fifteenth of the lunar calendar per month, and burn incense daily. We pooled the singular categories together to form successive frequencies to improve the statistical power of the corresponding stratums. We defined those who burn incense during festivals as occasional incense and defined those who burn incense on the first and fifteenth of the lunar calendar and daily incense as frequent incense. For cooking status, we had four categories: never cook at home, cook monthly, cook weekly and cook daily or more. We defined those who cook monthly and weekly as occasional cook, and cook daily or more as frequent cook. For wood fuel use for cooking, subjects chose between the two options of “yes” and “no”. Finally, for use of mosquito coils, we defined those who burn mosquito coils at least three times per week in the summer as “frequently using”, users who burn mosquito coils less than three times per week in the summer as “occasionally using”, and those who never burn mosquito coils as “never using”. In addition, other potential risk factors were also included and have been reported previously, such as the traditional Cantonese diet of salted fish, preserved vegetables, herbal tea, slow-cooked soup [35], alcohol and tea [34], as well as a family history of NPC in first-degree relatives [31].

T tests and Chi-square tests were used to characterize the case-control frequency distributions of the demographic information and potential risk factors for NPC. Multivariable unconditional logistic regression was used to evaluate the odds ratios (ORs) and corresponding confidence intervals (95 % CIs) after adjusting for the potential confounding factors of age (years, continuous variable), sex (male, female), education (high school or less, college or more), housing type (block, bungalow), cigarette smoking pack-years (never smoker, less than 20 pack-years, more than 20 pack-years), salted fish (less than monthly, monthly, weekly or more), preserved vegetables (less than monthly, monthly, weekly or more), tea (less than monthly, monthly, weekly or more), herbal tea (less than monthly, monthly, weekly or more), slow-cooked soup (less than monthly, monthly, weekly or more) and family history of NPC (no, yes). Linear-trend tests were used to evaluate the associations between continuous variables and NPC risk. Rothman’s additive interaction effect of tobacco smoking and incense burning was analyzed with the following equation: *S* = (OR_11_ − 1) / (OR_10_ + OR_01_ − 2). All of the statistical analyses were carried out using STATA 10.0 (Stata Corp, College Station, TX), and P-values less than 0.05 with two-sided tests were regarded as statistically significant.

## Results

Social-demographic characteristics of the study population and potential risk factors of NPC are described in Table 1. There was no significant difference in age (cases: 46.11 ± 10.99 vs. controls: 46.42 ± 11.74, *P* = 0.387) and sex distribution (*P* = 0.096) between the 1845 cases and 2275 controls. Significant differences between cases and controls were observed for cigarette smoking, with cases being more likely to be heavy smokers of more than 20 pack-years (31.25 % vs. 21.51 %). Compared with controls, cases tend to have less consumption of tea, herbal tea and slow-cooked soup (less than monthly frequency for tea: 35.96 % vs. 21.80 %; for herbal tea: 25.85 % vs. 18.96 %; for slow-cooked soup: 14.43 % vs. 3.55 %). They also may have more salted fish and preserved vegetables (weekly or more frequency for salted fish: 8.72 % vs. 5.44 %; for preserved vegetables: 13.10 % vs. 6.60 %). In addition, cases had a higher rate of family history of NPC than controls (16.51 % vs. 5.29 %).

**Table 1:** Characteristics of social-demographics and major risk factors of NPC cases and controls (%)

Variables	Cases (*n* = 1845)	Controls (*n* = 2275)	P*
Age, years	46.11 ± 10.99	46.42 ± 11.74	0.387
Sex			
Female	496 (26.88)	665 (29.23)	
Male	1373 (73.12)	1610 (70.77)	0.096
Education			
High school or lower	1558 (84.77)	1554 (68.52)	
College or above	280 (15.23)	714 (31.48)	<0.001
Living type			
Block	671 (37.68)	1070 (47.79)	
Bungalow	1110 (62.32)	1169 (52.21)	< 0.001
Cigarette smoking, pack-years			
Never-smoker	839 (45.75)	1190 (52.56)	
<20	406 (22.42)	406 (25.93)	
≥20	566 (31.25)	487 (21.52)	< 0.001
Tea			
Less than monthly	653 (35.96)	495 (21.80)	
Monthly	312 (17.18)	634 (27.92)	
Weekly or more	851 (46.86)	1142 (50.29)	< 0.001
Herbal tea			
Less than monthly	474 (25.85)	427 (18.96)	
Monthly	725 (39.53)	984 (43.69)	
Weekly or more	635 (34.62)	841 (37.34)	< 0.001
Slow-cooked soup			
Less than monthly	264 (14.43)	80 (3.55)	
Monthly	207 (11.32)	232 (10.30)	
Weekly or more	1358 (74.25)	1940 (86.15)	< 0.001
Salted fish			
Less than monthly	1472 (80.22)	2014 (89.15)	
Monthly	203 (11.06)	122 (5.40)	
Weekly or more	160 (8.72)	123 (5.44)	< 0.001
Preserved vegetables			
Less than monthly	1318 (71.67)	1961 (86.81)	
Monthly	280 (15.23)	149 (6.60)	
Weekly or more	241 (13.10)	149 (6.60)	< 0.001
Family history of NPC			
No	1537 (83.49)	2075 (94.71)	
Yes	304 (16.51)	116 (5.29)	< 0.001

*T-tests and Chi-Square tests were used to describe certain characteristics between cases and controls

As presented in Table 2, there is a positive association between incense burning and NPC risk. Compared with those who never use incense, an elevated risk was found in those who burn incense frequently, with the OR of 1.73 (95 % CI = 1.43, 2.09) after adjusting for the potential confounding factors described in the Methods section. Furthermore, there is a linear trend (P_*trend*_ <0.001) between incense frequency and NPC risk among occasional users and frequent users. In addition, wood fuel use was associated with NPC risk with an OR of 1.95 (95 % CI = 1.65, 2.31) compared with non-wood fuel users. However, there was no significant association between fumes of mosquito coil burning or cooking fumes and NPC risk (detailed information in Table 2).

**Table 2:** Association between household inhalants and nasopharyngeal carcinoma risk

Exposure factors	Case	Control	OR (95 % CI)^a^	*P*
Incense use frequency				
Never	287	572	1.00 (reference)	-
Occasionally	405	647	1.03 (0.83, 1.27)	0.812
Frequently	1130	1035	1.73 (1.43, 2.09)	< 0.001
P_*trend*_^b^				< 0.001
Mosquito coil use frequency				
Never	633	841	1.00 (reference)	-
Occasionally	671	816	1.03 (0.88, 1.22)	0.682
Frequently	497	574	0.97 (0.81, 1.17)	0.764
Cooking frequency				
Never	862	1041	1.00 (reference)	-
Less than daily	266	365	0.95 (0.77, 1.17)	0.643
Daily	712	858	1.04 (0.87, 1.23)	0.681
Wood stove use				
No	585	1135	1.00 (reference)	-
Yes	1260	1140	1.95 (1.65, 2.31)	< 0.001

^a^ORs (odds ratios) were adjusted for age (years, continuous variable), sex (male, female), education (high school or less, college or more), housing type (block, bungalow), cigarette smoking pack-years (never smoker, less than 20 pack-years, more than 20 pack-years), salted fish (less than monthly, monthly, weekly or more), preserved vegetables (less than monthly, monthly, weekly or more), tea (less than monthly, monthly, weekly or more), herbal tea (less than monthly, monthly, weekly or more), slow-cooked soup (less than monthly, monthly, weekly or more), family history of NPC (no, yes)

^b^Linear trends tests were performed by treating ordered categorical variables as continuous variables

Interestingly, we found a statistically significant additive interaction effect between heavy smokers of more than 20 pack-years and frequent incense burning for NPC risk (SI = 1.67; 95 % CI: 1.01, 2.76). Given that the association between tobacco smoking and NPC risk was discussed in our pioneering study [33], we did not concentrate on the role of cigarette smoking in NPC risk in this paper. As seen in Table 3, comparing those who were non-smokers and burned incense frequently with non-smokers who did not burn incense, the OR and 95 % CI was 1.83 (1.41, 2.38). The OR for those who smoke heavily and never burn incense compared with the same reference group was 1.76 (1.16, 2.68). Furthermore, there was a considerably higher risk among those who smoke more than 20 pack-years and use incense frequently, with an elevated OR and 95 % CI of 3.66 (2.65, 5.06). However, no significant addictive interaction effect was observed between wood fuel use and heavy smoking.

**Table 3:** Joint effects of inhalants and high exposure of cigarette smoking on nasopharyngeal carcinoma

Exposure factors	Never smokers			Ever smokers ≥20 pack years		
	Case	Control	OR (95 % CI)^a^	Case	Control	OR (95 % CI)^a^
Incense use						
Never	142	331	1.00 (reference)	72	101	1.76 (1.16, 2.68)
Frequent	489	506	1.83 (1.41, 2.38)	390	247	3.66 (2.65, 5.06)
Synergistic index^b^			SI = 1.67 (1.01, 2.76)			
Wood stove use						
No	265	629	1.00 (reference)	180	198	2.46 (1.80, 3.37)
Yes	574	561	2.30 (1.82, 2.90)	386	289	3.22 (2.39, 4.34)
Synergistic index^b^			SI = 0.81 (0.57, 1.13)			

^a^ORs (odds ratios) were adjusted for age (years, continuous variable), sex (male, female), education (high school or less, college or more), housing type (block, bungalow), salted fish (less than monthly, monthly, weekly or more), preserved vegetables (less than monthly, monthly, weekly or more), tea (less than monthly, monthly, weekly or more), herbal tea (less than monthly, monthly, weekly or more), slow-cooked soup (less than monthly, monthly, weekly or more), and family history of NPC (no, yes)

^b^The synergy index for household inhalant exposure and cumulative cigarette smoking pack-years

Similarly, we found a statistically significant additive interaction effect between wood fuel use and NPC family history on NPC risk (SI = 1.77; 95 % CI: 1.06, 2.96). As shown in Table 4, those who were wood fire users and had no NPC family history had a higher risk than non-wood fire users without a family history of NPC, ORs and 95 % CI of 1.94 (1.63 to 2.32). The OR for those who were non-wood fuel users and had no NPC family history compared with the same reference group was 3.67 (2.51 to 5.36). Furthermore, there was an obvious increased risk among those who were wood fuel users and had a family history of NPC with an elevated OR and 95 % CI of 7.39 (5.26, 10.37). However, no additive interaction effect was observed between incense use and NPC family history.

**Table 4:** Joint effects of inhalants and NPC family history on nasopharyngeal carcinoma

Exposure factors	Without NPC family history			Without NPC family history		
	Case	Control	OR (95 % CI)^a^	Case	Control	OR (95 % CI)^a^
Incense use						
Never	249	534	1.00 (reference)	38	23	3.44 (1.94, 6.10)
Frequent	932	937	1.72 (1.41, 2.10)	196	58	6.15 (4.32, 8.74)
Synergistic index^b^			SI = 1.63 (0.80, 3.30)			
Wood stove use						
No	491	1035	1.00 (reference)	91	57	3.67 (2.51, 5.36)
Yes	1046	1040	1.94 (1.63, 2.32)	213	59	7.39 (5.26, 10.37)
Synergistic index^b^			SI = 1.77 (1.06, 2.96)			

^a^ORs (odds ratios) were adjusted for age (years, continuous variable), sex (male, female), education (high school or less, college or more), housing type (block, bungalow), cigarette smoking pack-years (never smoker, less than 20 pack-years, more than 20 pack-years), salted fish (less than monthly, monthly, weekly or more), preserved vegetables (less than monthly, monthly, weekly or more), tea (less than monthly, monthly, weekly or more), herbal tea (less than monthly, monthly, weekly or more), and slow-cooked soup (less than monthly, monthly, weekly or more)

^b^The synergy index for household inhalant exposure and NPC family history

## Discussion

This is the first comprehensive and large-sample case-control study to unmask the association between household inhalants and NPC risk in southern China—one of the highest NPC risk areas in the world. We observed a significant association between frequent exposure to incense smoking and NPC risk. Interestingly, we found a significant additive interaction between exposure to frequent incense burning and cumulative cigarette smoking on NPC risk. This suggests that incense burning may not only associated with NPC independently but also increase the susceptibility of NPC risk jointly with other unfavorable factors, such as cigarette smoking. In addition, using wood fuel for cooking at home was also associated with elevated NPC risk and may co-contribute with NPC family history to NPC risk. This shows that wood combustion may have a combined effect with NPC family history for certain similar living environments or shared genetic backgrounds. However, no association between mosquito coil use and cooking fumes and NPC risk has been observed.

In our study, more than half of the subjects reported burning incense frequently at home (2165/4076 = 53.12 %). We observed that people who use incense frequently have about 70 % higher risk of NPC than those who never burn incense. The mechanism has been studied before. Incense burning emits several components similar to cigarette smoke and even exerts a higher genotoxicity on eukaryotic cells than tobacco smoke [36]. According to experimental studies, incense smoke released at least 4.5 times more particulate matter than an equal weight of cigarette smoke (45 mg/g vs. 10 mg/g) and caused an analogous amount of indoor pollution to tobacco smoke [37, 38]. The carcinogenic pollutants of benzenes, PAHs and 1,3-butadiene contained in incense may be involved in the development of cancer by reactivating the products that can cause DNA double-strand breakage, reduce base repair capacity, induce DNA adduct formation or trigger oxidative damage. In addition, a linear trend association (P_*trend*_ <0.001) was observed between incense frequency and NPC risk which indicated a long-term dose effect on NPC carcinogenesis. Similar findings were noted in previous studies conducted in Hong Kong. Sturton et al. reported a higher proportion of incense users among NPC patients compared to other cancer patients half a century ago [12]. Another study of 150 NPC patients and 150 controls in Hong Kong also revealed a positive association among people practicing Buddhism or paying homage to deities and having worship altars at home [10]. More recently, a hospital-based study of 352 cases and 410 controls in Hong Kong Chinese observed an increased NPC risk in females who burn incense daily with OR (95 % CI) = 2.49 (1.33, 4.66), but not in males [11]. However, the harmful effect of incense burning on NPC was observed among both females and males in our study and did not discriminate between genders (see Additional file 1: Table S1), which further strengthens the evidence that incense burning, like cigarette smoking, was a potential risk factor for NPC. However, the only existing population-based cohort study of 61,320 Singapore Chinese conducted between 1993 and 1998 with follow-up through 2005 found a null association between incense burning and NPC. Given that the single measurement of incense use was only detected at baseline between 1993 and 1998 and did not reassess at the end of follow-up in 2005, this could lead to the misclassification of subjects during the study.

The association between tobacco smoking and NPC risk has been confirmed. Our previous also demonstrated that cigarette smoking extracts can promote the activation of Epstein-Barr virus, which may be heavily involved in the occurrence and development of NPC [33]. More intriguingly, we found a significant additive interaction between the cigarette smoking and incense smoke for NPC risk with a synergistic index of 1.67. Similar findings were proposed by Tang et al. who showed a substantial elevated risk among smokers who use incense daily on lung cancer [39]. Smoking may induce chronic inflammation in the airways, which could cause reactive oxygen species (ROS) and DNA damage, and contribute to the interaction between tobacco smoking and incense smoke, ultimately facilitating the initiation and promotion of cancer progression [40–42].

There were 2399 subjects (58.28 %) who used wood fuel as the main fuel type for cooking or heating at home in our study. A study of 88 cases and 176 controls conducted in Guangxi, China, found that the use of wood fire was independently associated with NPC risk (OR = 6.4, *P* = 0.003) [29]. This is consistent with our results. We also found a significant increased NPC risk for daily cooking among wood fuel users (see Additional file 2: Table S2). A study conducted in North Africa found a null association between wood fire use and NPC risk during both childhood or adulthood [43]. Given that hereditary traits may play a role in NPC risk among different populations, the association between wood fuel use and NPC risk may depend on a population’s genetic background. Another Interesting founding was that wood fuel use for cooking co-contributed with NPC family history to increase NPC risk, with a synergistic index of 1.77, which shows that wood combustion may have a combined effect with NPC family history, this may because that family members live in certain similar living environments and share genetic backgrounds. Further studies are needed to confirm this association and to explore the detailed mechanisms of this combined effect.

However, there were some limitations in our study. First of all, recall bias and reporting bias were inevitable in the retrospective study. The association between household inhalants and NPC risk has not been widely known to the public, and subjects were asked to answer questionnaires about resident health lifestyles and not about cancer or disease research. Thus, even if these biases exist, null association would be achieved as both the cases and controls were influenced to the same degree. Second, hospital-based controls may cause selection bias. Controls from our study were healthy individuals from a hospital’s physical examination center. The prevalence of current tobacco smoking for males and females in our study was 48.4 and 1.1 %, respectively, which was quite close to that of adult tobacco use prevalence in the Global Adult Tobacco Survey (GATS) of China in the latest WHO survey, which found a prevalence of 52.9 % for males and 2.4 % for females (http://www.who.int/tobacco/surveillance/en_tfi_china_gats_factsheet_2010.pdf?ua=1). Thus, the representativeness of our controls was reliable. Third, we hardly can obtain some detailed information that may affect the inhalants for each person, such as ventilation conditions, burning type, and count or burning duration for every use. Given that there is no standard methodology to measure burning exposure because of the uniqueness of each household’s practices, it is difficult to evaluate the variance across studies.

## Conclusions

Our large-scale epidemiological study shows that incense smoke is not only a potential risk factor for NPC but also co-contributes with cigarette smoking to increase the risk of NPC in southern China. In addition, we found incense burning to be an independent risk factor, even among non-smokers. Moreover, wood combustion is another important risk factor and has a joint effect with NPC family history on NPC risk, while we have no evidence for an association between mosquito coils or cooking fumes and NPC risk in southern China. Our results suggest that eliminating or prohibiting incense burning and wood fire use at home is of meaningful public sanitation significance, especially in NPC endemic areas in China. Large-scale prospective cohort studies are needed to ascertain the robust causal association between household inhalants and NPC risk in South China.

## Additional files

Additional file 1: Table S1. Association between household inhalants and nasopharyngeal carcinoma risk, by sex. (DOC 43 kb) Additional file 2: Table S2. Association between cooking fumes and nasopharyngeal carcinoma risk among wood fire users. (DOC 29 kb)

## Abbreviations

95 % CI: 95 % confidence interval
NPC: nasopharyngeal carcinoma
SI: synergistic index
