# Supplementary material for: Household inhalants exposure and nasopharyngeal carcinoma risk: a large-scale case-control study in Guangdong, China
Source: BMC Cancer. 2015 Dec 29;15:1022. doi: 10.1186/s12885-015-2035-x (PMC4696254; doi:10.1186/s12885-015-2035-x)
Supplement: Additional file 2: Table S2. — Association between cooking fumes and nasopharyngeal carcinoma risk among wood fire users. (DOC 29 kb) [file 12885_2015_2035_MOESM2_ESM.doc]

Table S2. Association between cooking fumes and nasopharyngeal carcinoma risk among wood fire users.

| Cooking frequency | Case | Control | OR (95% CI)*^a^* | P |
| --- | --- | --- | --- | --- |
| Never | 543 | 525 | 1.00 (reference) |  |
| Less than daily | 176 | 172 | 1.11 (0.84,1.46) | 0.455 |
| Daily | 541 | 442 | 1.26 (1.01,1.58) | 0.039 |
| P*_trend_* |  |  |  | 0.039 |

*^a^* ORs (odds ratios) were adjusted for age (years, continuous variable), education (high school or less, college or more), housing type (block, bungalow), cigarette smoking pack-years (never smoker, less than 20 pack-years, more than 20 pack-years), salted fish (less than monthly, monthly, weekly or more), preserved vegetables (less than monthly, monthly, weekly or more), tea (less than monthly, monthly, weekly or more), herbal tea (less than monthly, monthly, weekly or more), slow-cooked soup (less than monthly, monthly, weekly or more), and family history of NPC (no, yes).
